# Tepid Modified Del Nido Cardioplegia in Adults Undergoing Cardiac Surgery: A Propensity-Matched Analysis

**DOI:** 10.21470/1678-9741-2020-0422

**Published:** 2022

**Authors:** Utkan Sevuk, Seyithan Dursun, Elif Sevgi Ar

**Affiliations:** 1 Department of Cardiovascular Surgery, Diyarbakir Gazi Yasargil Training and Research Hospital, Diyarbakir, Turkey.; 2 Department of Perfusion, Diyarbakir Gazi Yasargil Training and Research Hospital, Diyarbakir, Turkey.

**Keywords:** Cardioplegia, Cardiopulmonary Bypass, Heart Arrest, Induced, Myocardium, Constriction

## Abstract

**Introduction:**

Del Nido cardioplegia was reported to provide adequate myocardial protection and clinical outcomes with improved surgical flow in adult cardiac surgical procedures. And many clinicians have already modified the traditional formula. This study aims to investigate the efficacy and safety of tepid modified del Nido cardioplegia compared to cold blood cardioplegia in adult patients undergoing cardiac surgery.

**Methods:**

This retrospective study included one hundred consecutive adult patients undergoing cardiac surgical procedures using tepid modified del Nido cardioplegia. One hundred consecutive adult patients undergoing cardiac surgical procedures with cold blood cardioplegia were the control group. Propensity score matching yielded 89 modified del Nido and 89 cold blood cardioplegia patients.

**Results:**

There were no significant differences when comparing the two matched groups regarding the requirement for intraoperative defibrillation (*P*=0.36), postoperative peak troponin T levels (0.18), perioperative inotropic support (*P*=0.26), intra-aortic balloon pump requirement (*P*=0.62), and postoperative left ventricular ejection fraction at discharge (*P*=0.4) and on the sixth postoperative month (*P*=0.37). Mean cross-clamping time (*P*=0.005), cardiopulmonary bypass time (*P*=0.03), and total operation time (*P*=0.03) were significantly shorter in the del Nido group.

**Conclusion:**

Tepid modified del Nido cardioplegia may be a safe alternative to cold blood cardioplegia in adult patients undergoing cardiac surgical procedures.

**Table:** Abbreviations, acronyms & symbols

ACC	= Aortic cross-clamping
AF	= Atrial fibrillation
BC	= Blood cardioplegia
BMI	= Body mass index
CABG	= Coronary artery bypass grafting
CBC	= Cold blood cardioplegia
COPD	= Chronic obstructive pulmonary disease
CPB	= Cardiopulmonary bypass
CVE	= Cerebrovascular event
DM	= Diabetes mellitus
DNC	= Del Nido cardioplegia
EF	= Ejection fraction
ES	= Erythrocyte suspension
EuroSCORE	= European System for Cardiac Operative Risk Evaluation
GIS	= Gastrointestinal system
Hct	= Hematocrit
HL	= Hyperlipidemia
HS	= Hot shot
HT	= Hypertension
IABP	= Intra-aortic balloon pump
ICU	= Intensive care unit
LVEF	= Left ventricular ejection fraction
MDNC	= Modified del Nido cardioplegia
MI	= Myocardial infarction
PAD	= Peripheral arterial disease
RV	= Right ventricular

## INTRODUCTION

Single-dose del Nido cardioplegia (DNC) has been used widely in congenital heart surgery for more than 20 years. Single-dose DNC has been shown to be safe and effective in pediatric patients and to provide a safe period of cardiac arrest for up to 120 minutes^[1,2]^. In recent years, DNC has also been shown to provide safe and effective myocardial protection in adults^[3-7]^.

The original DNC consists of one part of fully oxygenated patient whole blood to four parts of Plasma-Lyte A (Baxter Healthcare Corporation, Deerfield, Illinois, United States of America) and is given anterogradely as a single dose and at a temperature of 8-12°C every 90 minutes^[1]^. The traditional formula has been modified by many clinicians^[8-12]^. In our protocol of modified del Nido cardioplegia (MDNC), cardioplegia was given at a blood to crystalloid ratio of 4:1 and at a tepid temperature (28°C) every 45 minutes.

The aim of this study is to evaluate the efficacy and safety of tepid MDNC compared to cold blood cardioplegia (CBC) in adult patients undergoing cardiac surgery. Although cold MDNC has been reported in the literature, to the best of our knowledge, this is the first study to evaluate the safety and efficiency of a single-dose tepid MDNC.

## METHODS

### Study Population and Design

This study was approved by our local Ethics Committee and complied with the requirements of the Declaration of Helsinki. We retrospectively reviewed the medical records of adult patients who underwent cardiac surgery under cardiopulmonary bypass (CPB) between January 2014 and December 2019. One hundred consecutive adult patients undergoing cardiac surgical procedures using MDNC and 100 consecutive adult patients undergoing cardiac surgical procedures with CBC were included in the study.

The exclusion criteria were as follows: patients who underwent emergency or salvage surgery as well as reoperative surgery, patients with recent acute coronary syndrome, patients who required inotropes/vasopressor support in the preoperative period, patients who had dialysis-dependent renal failure, patients who required a second period of aortic cross-clamping (ACC), and finally patients who underwent arrhythmia surgery using cryoablation due to its possible association with myocardial enzyme release.

### Operative Details

A standard general anesthesia protocol was used. The patients underwent minimally invasive procedures either with right infra-axillary minithoracotomy or ministernotomy under direct vision with central arterial and venous cannulation. The rest of the procedures were performed with a median sternotomy. In all procedures, CPB was established utilizing a shortened extracorporeal circulation circuit primed with Ringer's lactate solution and retrograde autologous priming to decrease hemodilution and the blood transfusion rate. Central cannulation was performed in all cases — a vent cannula was inserted into the left ventricle to prevent left ventricular distention. During CPB, the core temperature was cooled to 28°C. Concentrated fresh erythrocyte suspensions (≤ 7 days of storage) were added to the pump prime volume if required, to keep the hematocrit levels > 25% during CPB.

### Cardioplegia Strategy

Myocardial protection was obtained with the administration of either CBC or MDNC. No topical hypothermia was used. Antegrade cardioplegia was given at an aortic root pressure of 70-90 mmHg and delivered through the root cannula or by direct coronary ostial infusion. Retrograde cardioplegia was delivered at a pressure of 30-50 mmHg. A 500 mL of hot shot (HS) solution at 36°C was administered before the release of the aortic clamp in all cases. The compositions of both cardioplegia solutions are detailed in Table 1. The HS solution was identical in composition to the maintenance dose of CBC.

**Table 1:** Composition of cardioplegia solutions.

	Del Nido cardioplegia	Modified del Nido cardioplegia	Cold blood cardioplegia
Blood to crystalloid ratio	01:04	04:01	04:01
Base solution	1 liter of Plasma-Lyte	Saline (0.9% NaCl)	Saline (0.9% NaCl)
Mannitol	3.2 g/L	1 g/L	0
Magnesium sulphate	2 g/L	1 g/L	1 mg/mL (induction)
			0.5 mg/mL (maintenance)
NaHCO_3_	13 mEq/L	6 mEq/L	10 mEq/L
Potassium	26 mEq/L	30 mEq/L	30 mEq/L (induction)
			10 mEq/L (maintenance)
Lidocaine	130 mg	110 mg	0
Temperature of cardioplegia	+4°C	28°C	+4-8°C

### Cold Blood Cardioplegia Strategy

A 1-L induction CBC was given at a temperature of 4-8°C, and 500 mL of maintenance dose were given every 15-20 minutes. Combined antegrade-retrograde cardioplegia was delivered in patients who had coronary bypasses.

### Modified Tepid Del Nido Cardioplegia Strategy

The base solution for the traditional DNC is Plasma-Lyte A (Baxter Healthcare Corporation, Deerfield, Illinois, United States of America)^[1]^. The Plasma-Lyte A solution component was not used, and 0.9% NaCl was used as a base solution and mixed with the additives that comprise traditional DNC (Table 1). Cardioplegia was given at a blood to crystalloid ratio of 4:1 and at a tepid temperature (28°C). The induction and maintenance cardioplegias were delivered at 28°C (Table 1). The heart was arrested with an initial induction dose of 20 ml/kg with a maximum dose of 1000 mL for patients > 50 kgs. Subsequent doses (500 ml) were administered every 45 minutes. Cardioplegia was delivered anterogradely in all cases without any retrograde dosing.

### Primary Endpoints

The clinical manifestations of myocardial damage including postoperative peak troponin T levels, postoperative inotropic support requirement, defibrillation requirement after cross-clamp removal, postoperative left ventricular ejection fraction (LVEF) measured with transthoracic echocardiography before discharge (average of five days postoperatively) and at the sixth month of follow-up were primary endpoints of the study.

Measurement of troponin T was obtained in the postoperative period at 6, 12, 24, and 48 hours in both two groups.

### Secondary Endpoints

The secondary endpoints for the study were to evaluate postoperative clinical outcomes, CBP time, ACC time, and blood product use.

### Statistical Analysis

All statistical analyses were conducted using SPSS 22 software (IBM Corp. Released 2013. IBM SPSS Statistics for Windows, Version 22.0. Armonk, NY: IBM Corp.) All variables were investigated using visual (histograms, probability plots) and analytic methods (Kolmogorov-Smirnov test) to determine whether they were normally distributed. Continuous variables were reported as means and standard deviation for normally distributed variables and as medians and interquartile range for non-normally distributed variables. Categorical variables were presented using numbers and percentages. Comparison between the two groups was performed using the Chi-squared test or the Fisher's exact test for qualitative variables, the independent t-test for normally distributed continuous variables, and the Mann-Whitney U test for non-normally distributed continuous variables.

Propensity score matching analysis was performed to reduce the impact of selection bias and potential confounders secondary to non-randomization. The type of cardioplegic solution was used as a dependent binary variable, the following variables were included in a logistic regression model as independent variables to estimate the propensity scores: age, sex, body mass index, smoking, hypertension, diabetes mellitus, hyperlipidemia, chronic obstructive pulmonary disease, peripheral vascular disease, history of cerebrovascular events, the European System for Cardiac Operative Risk Evaluation II, preoperative renal dysfunction, preoperative atrial fibrillation (AF), preoperative LVEF, preoperative hematocrit levels, and type of surgery. Cases with propensity scores differing by > 0.05 were considered unmatched. Nearest neighbor matching without replacement was used to match MDNC and CBC patients using linear propensity scores. Propensity matching identified 89 matched pairs for analysis. P-values < 0.05 were considered to indicate statistical significance.

## RESULTS

### Demographics and Baseline Clinical Profile

The clinical characteristics of the CBC and MDNC groups before and after matching are shown in Table 2. There was no difference in the demographics, comorbidities, preoperative baseline, and clinical characteristics between the groups (Table 2).

**Table 2:** Baseline characteristics and comorbidities.

	Unmatched			Matched		
	MDNC (n=100)	CBC (n=100)	*P*-value	MDNC (n=89)	CBC (n=89)	*P*-value
Age (years)	57.9±13.7	58.1±10.4	0.9	59.1±13.5	57.8±10.6	0.5
Male, n (%)	56 (56)	53 (53)	0.67	47 (52.8)	47 (52.8)	1
BMI (kg/m^2^)	26.1 (21.9-31)	26,34 (23.9-30.3)	0.27	26.4 (22.1-31.1)	26.4 (23.8-30.3)	0.72
Smoking, n (%)	26 (26)	28 (28)	0.75	24 (27)	23 (25.8)	0.86
HT, n (%)	34 (34)	35 (35)	0.88	32 (36)	29 (32.6)	0.64
HL, n (%)	20 (20)	15 (18)	0.35	16 (18)	14 (15.7)	0.69
DM, n (%)	23 (23)	24 (24)	0.87	21 (23.6)	18 (20.2)	0.59
COPD, n (%)	26 (26)	24 (24)	0.74	21 (23.6)	21 (24.7)	0.86
PAD, n (%)	6 (6)	7 (7)	0.77	5 (5.6)	6 (6.7)	0.76
History of CVE, n (%)	2 (2)	2 (2)	1	2 (2.2)	1 (1.1)	1
Renal dysfunction, n (%)	3 (3)	2 (2)	1	3 (3.4)	1 (1.1)	0.62
EuroSCORE II	1,97 (1.5-3,15)	2.04 (1.49-2.93)	0.85	1.94 (1.51-3.16)	2.04 (1.46-3.17)	0.59
Preoperative EF	55 (40-55)	50 (45-55)	0.55	55 (42-55)	50 (40-55)	0.13
Preoperative AF, n (%)	15 (15)	16 (16)	0.84	14 (15.7)	15 (16.9)	0.84
Preoperative Hct (%)	39 (36-42)	40 (38-42)	0.18	39 (36-41.5)	40 (38-42.5)	0.06

Values are presented as mean ± standard deviation, median (interquartile range), or n (%). *P*<0.05 was considered statistically significant AF=atrial fibrillation; BMI=body mass index; CBC=cold blood cardioplegia; COPD=chronic obstructive pulmonary disease; CVE=cerebrovascular event; DM=diabetes mellitus; EF=ejection fraction; EuroSCORE=European System for Cardiac Operative Risk Evaluation; Hct=hematocrit; HL=hyperlipidemia; HT=hypertension; MDNC=modified del Nido cardioplegia; PAD=peripheral arterial disease

### Primary Endpoints in Matched Patients

No differences in requirement for intraoperative defibrillation (*P*=0.36), postoperative peak troponin T levels (*P*=0.18), perioperative inotropic support (*P*=0.26), intra-aortic balloon pump requirement (*P*=0.62), and postoperative LVEF at discharge (*P*=0.4) and on the sixth postoperative month (*P*=0.37) were observed between the patients within the MDNC and the CBC group.

### Secondary Endpoints in Matched Patients

Median ACC time (74 [55-94.5] *vs*. 90 [59-117.5] minutes; *P*=0.005), CPB time (109 [86-130] *vs*.126 [88.5-155] minutes; *P*=0.03), and operation time (195 [170-215] *vs*. 210 [170-235] minutes; *P*=0.03) were significantly shorter in the MDNC group. No significant difference in postoperative new AF development (*P*=0.14) was observed between the groups. Transfusion rates were similar between the groups (*P*=0.9). There were no significant between-group differences in other intraoperative and postoperative clinical characteristics in both matched and unmatched cohorts. Table 3 and Table 4 depict the intraoperative and postoperative data of both groups, respectively.

**Table 3:** Operative characteristics.

	Unmatched			Matched		
	MDNC (n=100)	CBC (n=100)	*P*-value	MDNC (n=89)	CBC (n=89)	*P*-value
Surgical procedures, n (%)						
Adult congenital heart disease	10 (10)	10 (10)		9 (10.1)	10 (11.2)	
Single valve	24 (24)	25 (25)		24 (27)	22 (24.7)	
Double valve	23 (23)	20 (20)		18 (20.2)	19 (21.3)	
Triple valve	11 (11)	10 (10)		8 (9)	8 (9)	
Valve surgery + CABG	9 (9)	8 (8)		6 (6.7)	7 (7.8)	
Aortic surgery	11 (11)	10 (10)		10 (11.2)	9 (10.1)	
Aortic surgery + valve surgery	6 (6)	7 (7)		6 (6.7)	5 (5.6)	
Aortic surgery + CABG	3 (3)	5 (5)		3 (3.4)	4 (4.5)	
Aortic surgery + CABG + valve surgery	3 (3)	5 (5)		5 (5.6)	5 (5.6)	
Minimally invasive	17 (17)	18 (18)	0.85	16 (18)	17 (19.1)	0.85
**ACC time (min)**	77 (57-96)	89.5 (59-117)	0.02	74 (55-94.5)	90 (59-117.5)	0.005
**Total CPB time (min)**	116 (88-133.7)	125.5 (89.2-153)	0.08	109 (86-130)	126 (88.5-155)	0.03
**Total operation time (min)**	200 (171.2-218.8)	210 (170-235)	0.06	195 (170-215)	210 (170-235)	0.03
**Defibrillation requirement, n (%)**	10 (10)	14 (14)	0.38	9 (10.1)	13 (14.6)	0.36
**Nadir Hct during CPB (%)**	26 (25-29)	26 (25-30)	0.15	27 (26-30)	26 (25-28.5)	0.06
**Transfused ES (units)**	0 (0-1)	0 (0-1)	0.74	0 (0-1)	0 (0-1)	0.9

Values are presented as mean ± standard deviation, median (interquartile range), or (%). *P*<0.05 was considered statistically significant ACC=aortic cross-clamping; CABG=coronary artery bypass grafting; CBC=cold blood cardioplegia; CPB=cardiopulmonary bypass; ES=erythrocyte suspension; Hct=hematocrite; MDNC=modified del Nido cardioplegia

**Table 4:** Postoperative outcomes.

	Unmatched			Matched		
	MDNC (n=100)	CBC (n=100)	*P*-value	MDNC (n=89)	CBC (n=89)	*P*-value
Intubation time (hours)	7 (6-8)	7 (6-8)	0.5	7 (6-8)	7 (6-9)	0.47
ICU stay (days)	1.49 ± 1.2	1.55 ± 1.3	0.74	1.45±1.14	1.57±1.38	0.87
Hospital stay (days)	5 (5-6)	5 (5-6)	0.6	5 (5-6)	5 (5-6)	0.48
Peak troponin T (ng/L)	337.16 ± 34.8	383.5 ± 37.8	0.2	375.16 ±32.9	382.4±39.1	0.18
Inotropic support, n (%)	11 (11)	16 (11)	0.3	9 (10.1)	14 (15.7)	0.26
IABP requirement, n (%)	3 (3)	3 (3)	1	1 (1.1)	3 (3.4)	0.62
Perioperative MI, n (%)	0	1	1	0	0	
CVE, n (%)	1 (1)	2 (2)	0.56	1 (1.1)	2 (2.2)	1
Respiratory failure requiring reintubation, n (%)	2 (2)	3 (2)	1	2 (2.2)	2 (2.2)	1
Pneumonia, n (%)	3 (3)	3 (3)	1	3 (3.4)	3 (3.4)	1
Postoperative new AF, n (%)	20 (20)	25 (25)	0.34	15 (16.9)	23 (25.8)	0.14
Re-exploration for bleeding, n (%)	2 (2)	3 (3)	0.65	2 (2.2)	3 (3.3)	1
Mediastinitis, n (%)	1 (1)	1 (1)	1	1 (1.1)	1 (1.1)	1
Wound infection, n (%)	3 (3)	4 (3)	1	3 (3.4)	3 (3.4)	1
Acute renal dysfunction, n (%)	2 (2)	2 (2)	1	2 (2.2)	2 (2.2)	1
GIS complications	0	0		0	0	
EF before discharge, n (%)	55 (45-55)	55 (45-55)	0.7	55 (45-55)	50 (45-55)	0.4
EF by the sixth postoperative month, n (%)	55 (48.7-55)	50 (45-55)	0.44	55 (50-55)	50 (45-55)	0.37
RV dysfunction, n (%)	0	0	1	0	0	1
In-hospital mortality, n (%)	2 (2)	2 (2)	1	1 (1.1)	2 (2.2)	1

Values are presented as mean ± standard deviation, median (interquartile range), or (%). *P*<0.05 was considered statistically significant. AF=atrial fibrillation; CBC=cold blood cardioplegia; CVE=cerebrovascular event; EF=ejection fraction; GIS=gastrointestinal system; IABP=ıntra-aortic balloon pump; ICU=ıntensive care unit; MDNC=modified del Nido cardioplegia; MI=myocardial infarction; RV=right ventricular

## DISCUSSION

In the present study, we found that the two groups were comparable in terms of intraoperative defibrillation requirement, postoperative peak troponin T levels, perioperative inotropic and IABP requirements, and postoperative LVEF at discharge and on the sixth postoperative month. MDNC was associated with reduced ACC, CPB, and operation times compared to the CBC group. The transfusion rate and clinical outcomes were similar in both groups. To our knowledge, this is the first study to report the safety of a single-dose tepid MDNC in adult patients.

### The Rationale For 4:1 Blood to Crystalloid Ratio

Blood cardioplegia (BC) has many advantages. Compared to crystalloid solutions, BC is rich in nutrients and both fatty acids and glucose, which are the primary source of energy for adenosine triphosphate in aerobic and anaerobic states, respectively. Thus, BC replenishes depleted energy stores during the ischemic arrest period^[13]^. Furthermore, BC provides endogenous buffers and endogenous antioxidants and has physiological rheological properties^[13]^. As compared to the crystalloid cardioplegia, BC decreases hemodilution and transfusion requirements^[13]^. While dissolved oxygen is the primary oxygen source in crystalloid cardioplegia, oxygen delivery capacity of blood is superior due to both hemoglobin and dissolved oxygen^[13]^. More oxygen is supplied during cardiac arrest and ischemic arrest period with BC^[13]^. Superior osmotic properties of BC reduces myocardial edema formation and subsequent compliance changes^[13]^. Compared to crystalloid cardioplegia, BC was shown to provide superior myocardial protection in particularly high-risk patients and energy depleted hearts^[13]^. Considering the advantages of BC, a 4:1 ratio of blood to crystalloid was utilized in our MDNC protocol.

### The Rationale for Tepid Temperature and Re-Dosing Intervals

Cardioplegia temperature is one of the mainstays of the myocardial protection strategy. Cardioplegia can be delivered as cold (4-10°C), tepid (27-30°C), or normothermic (34-37°C), each one having its own advantages and disadvantages^[14]^. The optimal temperature for cardioplegia has not yet been determined. Basal metabolism and oxygen consumption are reduced as a result of hypothermia. In addition to systemic hypothermia, selective myocardial hypothermia through cold cardioplegia further reduces the metabolism of the myocardium^[14]^. While hypothermia preserves the myocardium during cardiac arrest, hypothermia itself may have some detrimental effects on the myocardium, including negative impacts on ongoing enzymatic and cellular reparative processes, inhibition of ion pump activity and increased risk of myocardial edema, reduction in the therapeutic effects of some pharmacological drugs (such as additives) as a result of inhibition of various membrane receptors, increased plasma viscosity, and a decrease in red cell deformability^[14]^. Citing the hypothermic inhibition of myocardial enzymes, recovery of the myocardium may be delayed after cross-clamp removal^[14]^. The oxygen dissociation curve is shifted to the left due to an increased oxygen affinity of hemoglobin in cold alkalotic cardioplegia solution^[14]^. Thus, oxygen supply to the myocardium is diminished, and it has been found that the major oxygen source presented to the myocardium with hypothermic alkalotic BC is the oxygen dissolved in the solution^[14]^.

A significant part of the benefits of BC occurs at 37°C and are temperature dependent. These benefits may decrease with lower temperatures^[14]^. To get the highest benefit from the blood and to eliminate the deleterious effects of hypothermia, warm cardioplegia was introduced^[14]^. Warm cardioplegia is thought to provide the continuation of aerobic metabolism during the ischemic period, reduce the rewarming and perfusion time — and, consequently, the CPB time —, and eliminate the detrimental effects of systemic hypothermia^[14]^. Warm BC has been shown to result in improved metabolic and functional recovery of the myocardium in many experimental models^[14]^. On the other hand, higher temperature results in increased myocardial requirements, and it may be difficult to maintain complete electromechanical quiescence. The tolerance of myocardium to ischemic insult is reported to reduce in normothermic heart, and a normothermic myocardium was shown to be susceptible to ischemic injury in case of interruption or maldistribution of the cardioplegic solution^[14]^. Albeit the reports of decreased tolerance to ischemia with warm cardioplegia, there are studies reporting that a single dose of warm cardioplegia preserves the myocardium up to 40 minutes^[15-17]^. Minatoya et al.^[15]^ reported that intermittent warm BC provided 30 minutes of safe ischemia in patients who underwent coronary bypass surgery. Ghazy et al.^[16]^ showed that the protection of the first shot of warm cardioplegia might be > 20 minutes. Later, the same group reported that the time interval between the warm cardioplegic doses could be safely increased to > 35 minutes^[17]^.

Tepid cardioplegia was introduced to provide some benefits of warm cardioplegia while minimizing the deficits of warm cardioplegia and the adverse effects of cold cardioplegia^[14]^. Hayashida et al.^[18]^ reported that reducing the heart temperature from 37°C to 29°C did not change myocardial oxygen consumption, which suggests the preservation of mitochondrial function. Anaerobic lactate and acid release during the arrest were decreased with tepid cardioplegia. Myocardial function was preserved and, compared to cold cardioplegia, myocardial recovery was immediate. They concluded that myocardial protection was better with tepid cardioplegia than warm and cold cardioplegia^[18]^. In a study by Ramani et al.^[19]^, single-dose lidocaine containing 4:1 BC delivered at 20°C was shown to be safe and effective in preserving the myocardium.

Several factors influence the temperature of the myocardium, including the return of blood from pulmonary bronchial and mediastinal noncoronary collaterals, perfusate (systemic blood) temperature during partial CPB, operative lights, operating room temperature, and conduction of heat from the surrounding tissues. As shown in previous studies, the myocardial temperature rises during the clamping period after the induction of cold cardioplegia and approaches systemic temperature until the next dose of cardioplegia. Hence, hypothermia's myocardial protective effect decreases over time, particularly in patients who receive single-dose cardioplegia. In a study by Rao et al.^[20]^, patients were cooled down to 32°C and were given DNC at 4°C. They found that by 40 minutes, post-initial cardioplegia delivery transseptal probe temperature was approximately 25°C. Right ventricular temperature was even higher, approximating to 30°C by 40 minutes post-initial cardioplegia^[20]^. In a study by Momin et al.^[21]^, patients received single-dose DNC and were systemically cooled to 32°C. Right atrial temperatures were > 25°C at 30 minutes after induction. Boldt et al.^[22]^ reported rewarming of the heart to approximately 25°C 30 minutes after the induction with cold (4°C) cardioplegic solution. Topical cooling was used, and patients were cooled down to 34°C in this study. Daily et al.^[23]^ reported myocardial rewarming from 18°C to 22°C within 20 minutes after the delivery of cold cardioplegic solution. Patients were cooled down to 28°C in this study. Okamoto et al.^[24]^ found an approximately 10°C increase in myocardial temperature after 30 minutes of an interval before the second dose of CBC under normothermic CPB. The hearts were rewarmed to approximately 25°C in this study. All these studies show that hypothermia is diminished as one of the main protecting factors of cold cardioplegia over time, particularly in patients receiving single-dose cardioplegia. After the induction dose, the heart remains warm for about 45 minutes of 90 minutes in patients receiving single-dose cardioplegia. Considering that these single-dose cardioplegia strategies were shown to be safe in patients with prolonged ischemic period^[5]^, we came to the conclusion that 45 minutes of ischemic period at a tepid temperature with a single-dose cardioplegia should be safe and could benefit from the advantages of tepid cardioplegia.

### Modified Del Nido Cardioplegia in Clinical Use

Previous studies demonstrated non-inferior or better myocardial protection in various cardiac surgical procedures with the use of DNC, including complex cardiac surgical procedures with prolonged ACC time^[3-5]^. Nevertheless, only few studies evaluated the safety of MDNC in adults undergoing cardiac surgery. Several modifications of DNC have been reported, including the base solution, blood to crystalloid ratio, temperature, re-dosing interval, and constituents^[8-12]^. Yammine et al.^[8]^ reported lidocaine containing ''modified del Nido" solution, which was compared with the whole standard BC in adult cardiac surgical procedures. MDNC was given at a blood to crystalloid ratio of 8:1. Their MDNC contained 0.9% NaCl as carrier, 8 mEq/L magnesium sulfate 50%, 30 mEq/L potassium chloride, and 100 mg/L lidocaine 1%. Magnesium sulfate and potassium chloride additive quantities were different from the traditional solution, and MDNC did not contain sodium bicarbonate and mannitol. Patients requiring cross-clamping time > 60 minutes were re-dosed with standard whole BC solution. There was no data regarding the temperature of the MDNC. They found that lidocaine containing cardioplegia may be a safe alternative in adults when administered for the first 60 minutes of ACC. Stamou et al.^[10]^ modified the traditional DNC by removing the crystalloid portion and using whole blood microplegia as the base solution. DNC additives were administered in the whole blood. They investigated the safety of MDNC in high-risk patients undergoing cardiac surgery and compared with low-risk patients undergoing cardiac surgery with the same cardioplegia protocol. The induction and maintenance cardioplegias were delivered at 6°C. Cardioplegia was repeated every 90 minutes. A single dose of MDNC contained 24 mEq/Lt potassium chloride, 6 mL of 2% lidocaine, 2.5 g/L of 25% mannitol, 2.7 g of 50% magnesium sulfate, 8.6 mEq/L of sodium bicarbonate, and 970 mL of oxygenated blood (Plasma-Lyte was removed). They reported that MDNC was safe in high-risk patients undergoing cardiac surgery and in prolonged operations. The same group compared their MDNC strategy with lidocaine containing CBC^[9]^. They found comparable clinical outcomes. Kantathut et al.^[11]^ compared MDNC with standard CBC in adult patients undergoing cardiac surgery. They removed Plasma-Lyte-A and used lactated Ringer’s solution as the carrier. Their MDNC contained traditional additives with the same amount and was delivered 1:4 with one part of oxygenated pump blood to four parts of cardioplegia solution. The delivery temperature was at 4°C and it was repeated every 90 minutes. They concluded that compared to BC, MDNC provided either similar or superior myocardial protection. Gallo et al.^[12]^ reported the successful use of a complementary dose of 4:1 (blood:crystalloid) cold MDNC in four patients during donor heart implantation. The normosol-R solution was used as a carrier. The modified solution contained 35 mEq/Lt potassium chloride, which is higher than the traditional quantity. Other additive quantities were identical to traditional DNC.

While providing appropriate myocardial protection is indispensable, reducing ACC and CPB times is also important to improve the perioperative outcomes. A single-shot of cardioplegia was reported to reduce ACC, CPB, and operative times while improving surgical flow^[3-5]^. Each additional cardioplegia dosing disrupts the surgical flow and potentially increases ACC, CPB, and operative times, notably in patients who receive multidose cardioplegia. We have found that MDNC was associated with reduced ACC and CPB times compared to the CBC group. However, this difference did not translate into improved clinical outcomes.

### Limitations

This study has several limitations. This is a single-center retrospective study; thus, randomization and blinding were not possible. Our study comprised low-risk patients; hence, our results cannot be generalized to a high-risk patient population. We compared MDNC with CBC; therefore, our results may not be generalizable to other modifications of DNC and other cardioplegic solutions.

## CONCLUSION

Our study revealed that a single shot of tepid MDNC might be a safe alternative to CBC in adults undergoing cardiac surgical procedures. Nevertheless, our results may not be generalizable to other modifications of DNC. Further investigations should be performed to clarify the maximum tolerable ischemic time with a single dose of tepid MDNC.
